# Improvement of the catalytic efficiency of a hyperthermophilic xylanase from *Bispora* sp. MEY-1

**DOI:** 10.1371/journal.pone.0189806

**Published:** 2017-12-18

**Authors:** Xiaoyu Wang, Fei Zheng, Yuan Wang, Tao Tu, Rui Ma, Xiaoyun Su, Shuai You, Bin Yao, Xiangming Xie, Huiying Luo

**Affiliations:** 1 College of Biological Sciences and Biotechnology, Beijing Forestry University, Beijing, People's Republic of China; 2 Key Laboratory for Feed Biotechnology of the Ministry of Agriculture, Feed Research Institute, Chinese Academy of Agricultural Sciences, Beijing, People's Republic of China; Weizmann Institute of Science, ISRAEL

## Abstract

Extremophilic xylanases have attracted great scientific and industrial interest. In this study, a GH10 xylanase-encoding gene, Xyl10E, was cloned from *Bispora* sp. MEY-1 and expressed in *Pichia pastoris* GS115. Deduced Xyl10E shares the highest identities of 62% and 57% with characterized family GH10 xylanases from *Talaromyces leycettanus* and *Penicillium canescens* (structure 4F8X), respectively. Xyl10E was most active at 93 to 95°C and pH 4.0, retained more than 75% or 48% of the initial activity when heated at 80°C or 90°C for 30 min, respectively, and hardly lost activity at pH 1.0 to 7.0, but was completely inhibited by SDS. Two residues, A160 and A161, located on loop 4, were identified to play roles in catalysis. Mutants A160D/E demonstrated higher affinity to substrate with lower *K*_*m*_ values, while mutants A161D/E mainly displayed elevated *V*_*max*_ values. All of these mutants had significantly improved catalytic efficiency. According to the molecular dynamics simulation, the mutation of A160E was able to affect the important substrate binding site Y204 and then improve the substrate affinity, and the mutation of A161D was capable of forming a hydrogen bond with the substrate to promote the substrate binding or accelerate the product release. This study introduces a highly thermophilic fungal xylanase and reveals the importance of loop 4 for catalytic efficiency.

## Introduction

Xylan is the second most abundant polysaccharide after cellulose. To digest polymerized xylan, acid, alkaline, and enzymatic methods have been widely used. Enzymatic hydrolysis is more favorable due to the high efficiency and environmentally friendly characteristics. Complete hydrolysis of xylan requires the synergetic action of several xylanolytic enzymes, including β-1,4-xylanases, β-D-xylosidases, α-L-arabinofuranosidases, α-glucuronidases, acetyl xylan esterases, and feruloyl esterases. Of them, β-1,4-xylanase plays a crucial role in breaking the backbones of xylan. According to the Carbohydrate-Active enZymes database (CAZy; http://www.cazy.org/), xylanases are classified into glycoside hydrolase (GH) families 5, 7, 8, 10, 11 and 43, and those from GH10 and GH11 are most widely studied [1]. GH11 xylanases fold into a β-jelly roll structure, while those of GH10 are TIM-barrel folds.

Xylanases have potential applications in various industries, for instance commercial food production, animal feed, baking, fruit juice clarification, pulp biobleaching and bioconversion [2–5]. According to the industrial requirements, the thermophilic and acidophilic enzymes are specifically used in the bioconversion process, feed and brewing fields [6]. For example, the bioconversion process involves steam explosion and acidic pretreatments prior to enzyme treatment [7, 8]. Since the enzymes cannot be present in steam explosion or strongly acidic (and high temperature) conditions, the enzymes have to be added at a later process stage. Xylanase as a feed additive must withstand the pelleting temperature (70–90°C) and adapt to digestive tract pH (4.8) [9]; the brewing industry requires acidic thermostable xylanase for persistent hydrolysis at 70°C [10].

Although most thermophilic xylanases of GH10 demonstrate a temperature optimum from 60 to 80°C [11–14], a few exceptions have temperature optima up to 105 and 110°C [15–16]. For industrial purposes, catalytic efficiency is another important enzyme characteristic for improvement. Chimeric xylanase CXC-X4,5 of GH10 had a catalytic efficiency of almost 30-fold higher than the wild-type [17], fusion xylanase with a xylan-binding domain showed catalytic improvement on insoluble substrate [18], and a single mutation H179F increased the catalytic efficiency of XynA from *Geobacillus stearothermophilus* by 2.5-fold [19]. In the present study, a hyperthermophilic xylanase was identified in *Bispora* sp. MEY-1, and site-directed mutagenesis was conducted to improve its catalytic efficiency.

## Material and methods

### Strains, media, vectors and chemicals

*Bispora* sp. MEY-1 CGMCC 2500 from the China General Microbiological Culture Collection Center (Beijing, China) was grown in PDB medium at 30°C. *Escherichia coli* Trans1-T1 (Tiangen, Beijing, China) was used for gene cloning and sequencing. Vector pPIC9 and *Pichia pastoris* GS115 from Invitrogen (Carlsbad, CA) were used for heterologous expression. Beechwood xylan was purchased from Sigma-Aldrich (St. Louis, MO). The DNA purification kit from OMEGA (Norcross, GA), KOD neo Plus polymerase from TOYOBO (Osaka, Japan), restriction enzymes from TaKaRa (Otsu, Japan), and total RNA isolation system kit and T4 DNA ligase from Promega (Madison, WI) were purchased. All chemicals were of analytical grade and commercially available.

### Cloning of the cDNA gene

The draft genome of *Bispora* sp. MEY-1 was sequenced and annotated with CAZy database. The full-length GH10 xylanase gene, *xyl10E*, was then identified. The total RNA was extracted from the 3-day-old mycelia and purified using the Promega SV Total RNA Isolation System. cDNAs were then synthesized *in vitro* using the ReverTra Ace-a-™ kit (TOYOBO). Specific primers were designed based on the known DNA sequence (S1 Table) and used to amplify the DNA fragment. The PCR product with the appropriate size was digested with *SnaB*I and *Not*I and ligated into the *SnaB*I-*Not*I-digested pPIC9 vector to construct recombinant plasmid pPIC9-*xyl10E*.

### Sequence analysis

BLASTx program (http://www.ncbi.nlm.nih.gov/BLAST/) and Clustal Omega (http://www.ebi.ac.uk/Tools/msa/clustalo/) were used to analyze the Xyl10E sequence and align the nucleotide and amino acid sequences, respectively. The multiple sequence alignment results were examined by ESPript 3.0 (http://espript.ibcp.fr/ESPript/cgi-bin/ESPript.cgi). SignalP4.0 server (http://www.cbs.dtu.dk/services/SignalP/) was used to predict the signal peptide. ProtParam tool (http://web.expasy.org/protparam/) was used to predict the molecular mass and theoretical isoelectric point (*p*I) of the mature protein.

### Heterologous expression and purification of Xyl10E

The recombinant plasmid pPIC9-*xyl10E* was linearized by *Bgl*II and then transformed into *P*. *pastoris* GS115 competent cells by electroporation. Protein expression was carried out as previously reported [20]. Positive transformants were screened via enzymatic activity assays in shake tubes, and the transformant with the highest xylanase activity was fermented in 1-L Erlenmeyer flasks.

The cultures were centrifuged to remove undissolved materials, and the culture supernatants were then concentrated by a Viva flow 200 ultrafiltration membrane (cut-off 10 kDa; Vivascience, Hannova, Germany) as previously reported [20]. The crude enzyme was dialyzed with buffer A (20 mM citric acid-Na_2_HPO_4_; pH 7.5) at 4°C overnight and loaded onto a HiTrap Q Sepharose XL 5 mL FPLC column (GE Healthcare, Uppsala, Sweden) equilibrated with buffer A. A linear gradient of buffer B (1 M NaCl dissolved in the buffer A) was used to elute the proteins. Fractions exhibiting enzyme activities were pooled and assayed by sodium dodecyl sulfate-polyacrylamide gel electrophoresis (SDS-PAGE). The purified Xyl10E was deglycosylated by endo-β-*N*-acetylglucosaminidase H (Endo H) (New England Biolabs, Hitchin, UK) at 37°C overnight. The protein concentration was determined using the Lowry method [21], with bovine serine albumin as the standard.

### Biochemical characterization of purified recombinant Xyl10E

The standard xylanase activity assay was performed at 95°C for 10 min in citric acid-Na_2_HPO_4_ (pH 4.0) containing 1.0% (w/v) beechwood xylan. The reducing sugar was determined using the 3,5-dinitrosalicylic acid (DNS) method [22]. The optimal pH was determined at 95°C in the range of pH 1.5 to 6.0, the optimal temperature was determined by performing the activity assay at the optimal pH from 40 to 95°C, and the assay of 98.5°C was determined in a bath of boiling water. The pH stability was determined by pre-incubating the enzyme (approximately 10 μg/mL) at 37°C for 1 h in buffers of pH 1.0 to 9.0 (glycine-HCl for pH 1.0–2.5, citric acid-Na_2_HPO_4_ for pH 2.5–8.0 and glycine-NaOH for pH 8.0–9.0), and the thermostability was monitored by incubating the enzyme (approximately 30 μg/mL) at 80°C or 90°C for 1 h. Residual activities were then measured at 95°C and pH 4.0 for 10 min.

Kinetic assays were performed at 95°C for 5 min in citric acid-Na_2_HPO_4_ (pH 4.0) with 0.5–10 mg/mL beechwood xylan as the substrate. The kinetic values were determined using the Michaelis-Menten model with the GraphPad Prism version 5.01 (La Jolla, CA). Three independent experiments were repeated, and each experiment contained three replicates.

The effect of different metal ions and chemical reagents on the Xyl10E activity was determined in the presence of 5 mM of NaCl, KCl, LiCl, MgSO_4_, CaCl_2_, HgSO_4_, AgNO_3_, ZnSO_4_, FeCl_3_, NiSO_4_, CuSO_4_, SDS, β-mercaptoethanol and EDTA. The reaction systems without any chemical addition were used as the control. All enzyme measurements were done in triplicate.

### Homology modelling and structure analysis

Modeller 9.13 was used to build the homology model of Xyl10E with Amber 14 package to process energy minimization. Pymol 0.99rc was employed to visualize the homology model and structure alignment. The catalytic amino acid residues and amino acid frequency in the Swiss-Prot database were predicted by HotSpot Wizard [23] (http://loschmidt.chemi.muni.cz/hotspotwizard/). Amino acid interactions were predicted by Residue Interaction Network Generator (RING) web server [24] (http://protein.bio.unipd.it/ring).

### Mutagenesis

Both A160 and A161 of Xyl10E were substituted with Asp (D) or Glu (E) individually to create four mutant enzymes: A160D, A160E, A161D, and A161E. The enzyme nomenclature indicates the type and location of the substitution; for example, A160D means that A160 was changed to D160. The site-directed mutants were generated by overlap PCR with KOD neo Plus polymerase. Mutants were generated by specific primers, as shown in S1 Table. Heterologous expression and purification of mutant enzymes were carried out as described in the section “Heterologous expression and purification of Xyl10E”.

### Molecular dynamics (MD) simulation

MD simulation was carried out with Amber 14 package at a temperature of 330 K for a 20-nanosecond process (Time step was 2 femtoseconds) with force fields ff99SB, Glycam_06j-1, and GAFF. Solvate model was TIP3P with a box of truncated octahedron. The closest distance between protein atoms and the periodic box was set to 1.2 nm. Trajectory data were analyzed with CPPTRAJ software [25].

## Results

### Gene cloning and sequence analysis

The cDNA of *xyl10E* was successfully amplified by specific primers Xyl10EF and Xyl10ER (S1 Table). It consists of 1,137 bp that codes for a polypeptide of 378 amino acid residues. The first N-terminal 18 amino acid residues were predicted to be a signal peptide, and the mature Xyl10E has a calculated molecular mass of 39.9 kDa. Deduced Xyl10E showed an identity of 100% to an uncharacterized xylanase from *Acidomyces richmondensis* (KXL49610.1), of 62% to a characterized GH10 xylanase from *Talaromyces leycettanus* (GenBank accession no. KX228205) [11], and of 57% to the structure-resolved xylanase from *Penicillium canescens* (PDB no. 4F8X).

### Expression and purification of the recombinant protein

The cDNA fragment coding for the mature Xyl10E was amplified by PCR with primers Xyl10ESnabF and Xyl10ENotR (S1 Table) and successfully expressed in *P*. *pastoris* GS115. After methanol induction, positive transformants showed detectable xylanase activities, and the transformant with the highest activity was fermented at a large-scale. Crude Xyl10E was then purified to electrophoretic homogeneity with a molecular mass higher than the calculated value (39.9 kDa). After Endo H treatment, the enzyme migrated as a single band with an apparent molecular weight of approximately 40 kDa, which is in line with its calculated value (S2 Fig).

### Biochemical properties of Xyl10E

**Fig 1:**
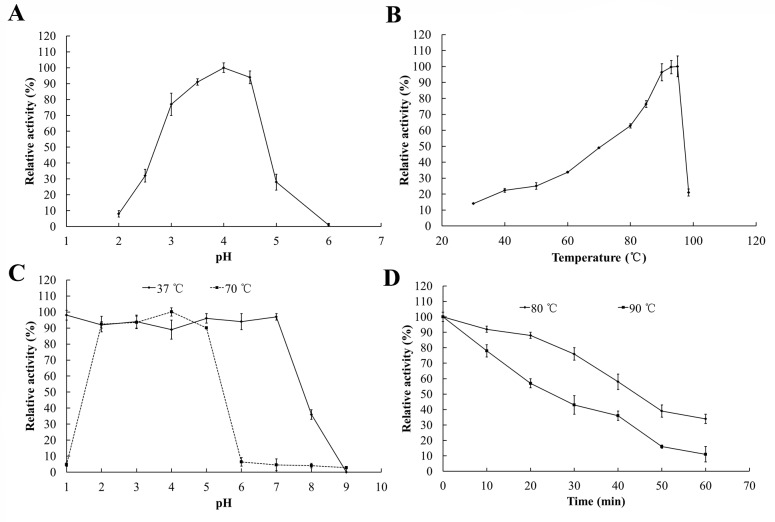
Characterization of the purified recombinant Xyl10E. (A) pH-activity profile. (B) Temperature-activity profile. (C) pH stability. (D) Thermostability.

Beechwood xylan was used as the substrate for enzyme assay. Xyl10E was active over an acidic pH scale ranging from 2.0 to 6.0 and a broad temperature range from 30 to 98.5°C, with the maximum activity at pH 4.0 (Fig 1A) and 93‒95°C (Fig 1B). The pH stability of Xyl10E was assayed at pH 1.0‒9.0 and 37°C or 70°C. After 1-h incubation, the enzyme retained more than 88% activity at pH 1.0 to 7.0 and 37°C and more than 90% activity at pH 2.0 to 5.0 and 70°C (Fig 1C), respectively. Xyl10E demonstrated excellent thermostability at high temperatures, retaining more than 75% and 48% after incubation at 80°C and 90°C for 30 min (Fig 1D), respectively. Moreover, Xyl10E displayed strong resistance to all tested metal ions and chemical reagents except for SDS (Table 1). The enzymatic activity was enhanced 1.3-fold by β-mercaptoethanol.

**Table 1:** Effects of metal ions or chemical reagents (5 mM) on Xyl10E activity.

Chemicals	Relative activity (%)^a^	Chemicals	Relative activity (%)
Control	100.0 ± 0.4	Ni^2+^	109.2 ± 2.8
K^+^	98.3 ± 0.6	Cr^3+^	107.1 ± 2.2
Mg^2+^	104.0 ± 9.7	Cu^2+^	90.6 ± 1.3
Na^+^	79.7 ± 10.5	Zn^2+^	94.2 ± 0.4
Ca^2+^	95.1 ± 2.1	Fe^3+^	108.9 ± 1.2
Pb^2+^	77.8 ± 1.7	β-Mercaptoethanol	130.2 ± 3.3
Ag^+^	83.6 ± 4.6	EDTA	83.1 ± 1.9
Mn^2+^	100.6 ± 8.7	SDS	0

^a^ Data are shown as mean ± SD (n = 3); the control had no chemical addition.

In addition, the specific activity, *V*_*max*_, *K*_*m*_ and *k*_*cat*_/*K*_*m*_ values of Xyl10E were determined to be 537 ± 13 U/mg, 692 ± 32 U/mg, 1.33 ± 0.21 mg/mL and 351 ± 56 mL/s/mg, respectively (Table 2).

**Table 2:** Kinetics parameters of Xyl10E and its mutants.

Enzyme	*K*_*m*_ (mg/mL)	*V*_*max*_ (U/mg)	*k*_*cat*_ (1/s)	*k*_*cat*_/*K*_*m*_ (mL/s/mg)	Specific activity (U/mg)
Wild-type	1.33 ± 0.21	692 ± 32	460 ± 21	351 ± 56	537 ± 13
Xyl10E_A160D	0.69 ± 0.08	606 ± 35	412 ± 26	587 ± 70	542 ± 18
Xyl10E_A160E	0.63 ± 0.11	593 ± 24	436 ± 16	638 ± 83	546 ± 22
Xyl10E_A161D	1.04 ± 0.04	994 ± 19	663 ± 13	625 ± 12	704 ± 36
Xyl10E_A161E	1.26 ± 0.17	964 ± 12	642 ± 8	516 ± 44	631 ± 32

### Homology modelling and structure analysis

Homology modelling of Xyl10E and its complex with ligand were conducted using the structure-resolved xylanases 4F8X and 1UQY as templates. After energy minimization by Amber 14 with steepest decent methods (1,000 steps) and conjugate gradient methods (100 steps), the modelled receptor-ligand complex was used for analysis. Modelled Xyl10E contained a typical TIM-barrel structure. HotSpot Wizard analysis identified E157 and E268 as the catalytic residues.

In a previous study, site-directed mutagenesis of G149D in *Tl*Xyn10A [11] introduced a hydrogen bond with a substrate and therefore enhanced the catalytic performance. Multiple sequence alignment (S3 Fig) indicated that Xyl10E has Ala at position 160 and 161 (corresponding to N148 and G149 of *Tl*Xyn10A). These sites located on loop 4 were then selected for mutagenesis.

### Mutagenesis

The four mutant enzymes were characterized and compared with the wild type (Table 2). Mutants A160D and A160E demonstrated greater substrate affinity (decreased *K*_*m*_ values of 1.9-fold) but slightly lower *V*_*max*_ and *k*_*cat*_ values, while mutant A161D had stronger substrate affinity, and both A161D and A161E demonstrated much higher *V*_*max*_ and *k*_*cat*_ values (1.4-fold higher than wild-type). As a result, the catalytic efficiency and specific activity were improved 1.5–1.8-fold and up to 1.3-fold, respectively (Table 2). Moreover, it can be concluded that too much increase in the enzyme affinity to the substrate (lower *K*_*m*_) may not always be beneficial to the enzyme efficiency, as seen in the mutations at 160 (see Table 2). Too much increase in the enzyme affinity may cause the product release to suffer.

### MD simulation analysis

**Fig 2:**
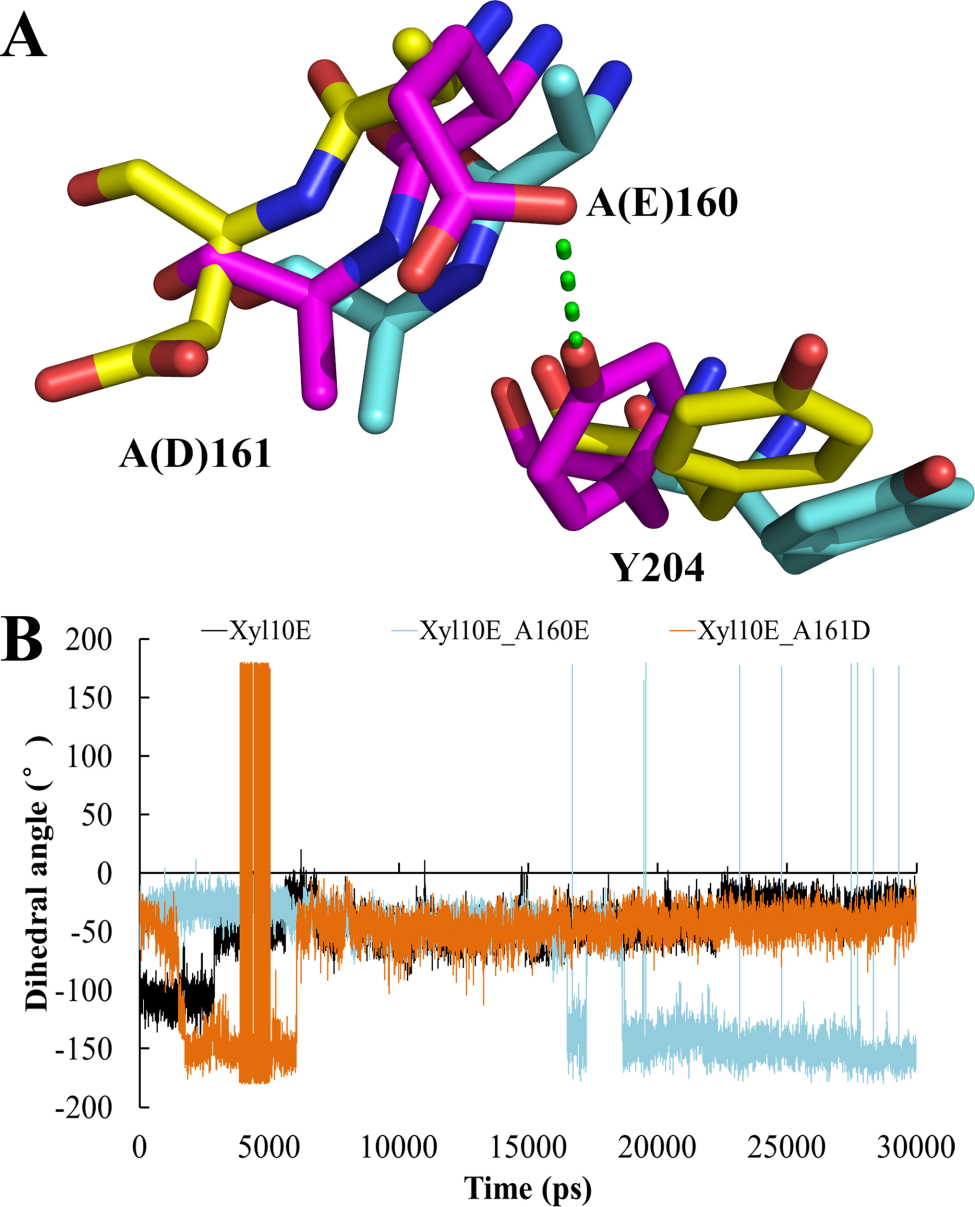
Analysis of the key residues in Xyl10E and its mutants. (A) Structure alignment of Xyl10E (yellow) and its mutants Xyl10E_A160E (blue) and Xyl10E_A161D (orange). Residues 160, 161, and 204 are indicated with sticks. Hydrogen bonds are indicated with green dashes. (B) Dihedral angles formed by the atoms N, C_β_, C_α_, and C_δ1_ of Y204 of Xyl10E and its mutants.

**Fig 3:**
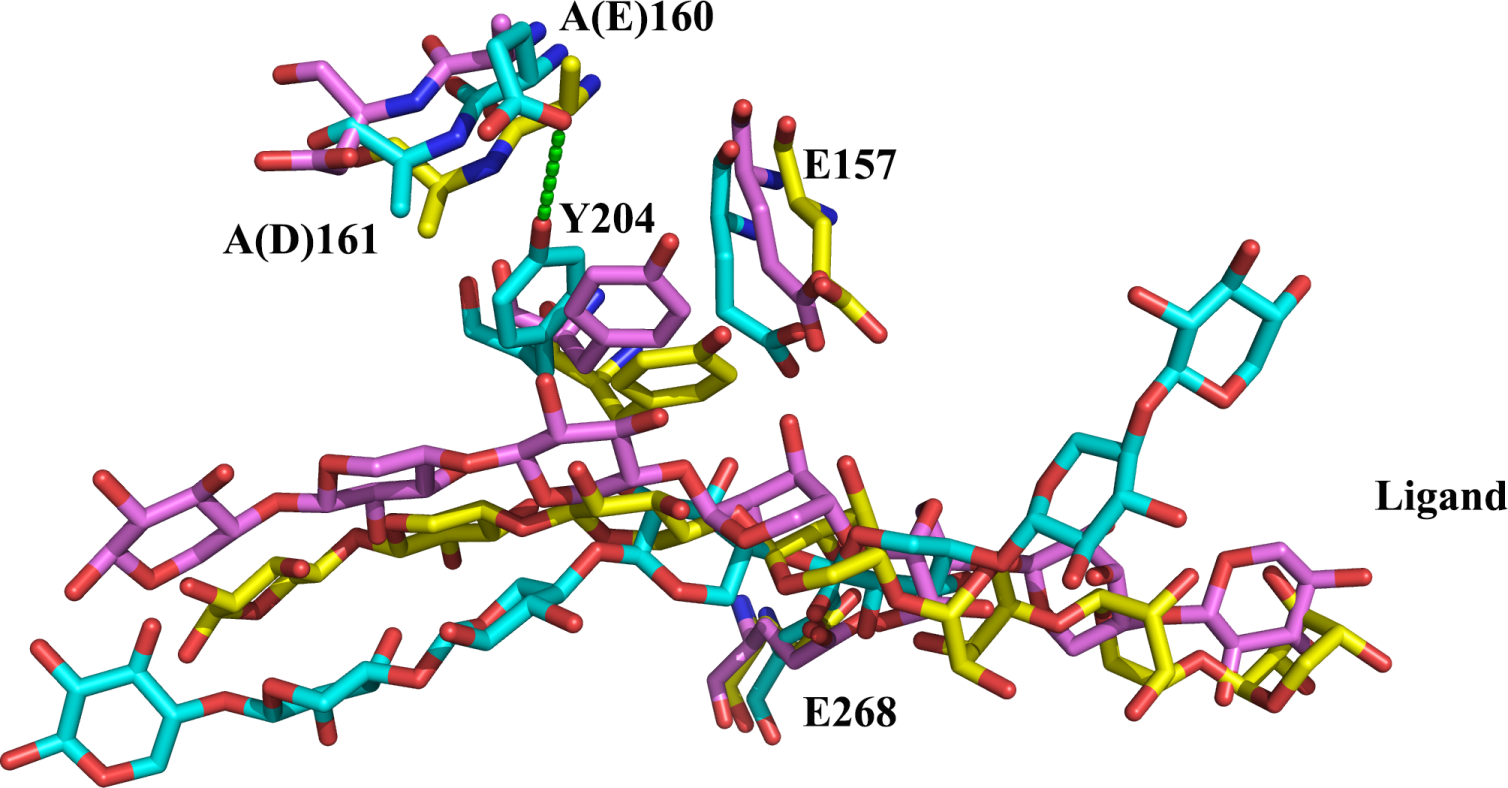
Structure alignment of the last frames of Xyl10E, Xyl10E_A160E and Xyl10E_A161D from the 30 ns MD trajectories. The key residues, including catalytic sites (E157 and E268), A(E)160, A(D)161, Y204, ligands of Xyl10E (yellow), Xyl10E_A160E (cyan), and Xyl10E_A161D (purple) are displayed by stick and ball models.

In order to investigate the subtle differences between wild type and mutants, MD simulation was carried out with Amber 14 package. As shown by the MD trajectories (Fig 2A), Y204, the key substrate binding residue (subsite +1) [26], demonstrated the same conformations of almost paralleled aromatic plates in Xyl10E and Xyl10E_A161D, but showed conformational difference in mutant Xyl10E_A160E by forming a hydrogen bond with the side chain of E160. Further analysis of the 30 ns trajectories indicated that the dihedral angles formed by the atoms N, C_β_, C_α_ and C_δ1_ of Y204 changed from ~ −50° (for wild-type and Xyl10E_A161D) to ~ −150° (for Xyl10E_A160E) (Fig 2B), confirming that the conformation of Y204 in Xyl10E_A160E was entirely different from that in Xyl10E and Xyl10E_A161D. This conformational change of Y204 (subsite +1) may account for the improved substrate affinity of Xyl10E_A160E. Moreover, significant differences were also identified in their ligand binding profiles (Fig 3). When exporting the last conformations of Xyl10E and its two mutants from the trajectories, Y204 of Xyl10E and Xyl10E_A161D formed hydrophobic interaction with the ligand, while Y204 of Xyl10E_A160E was far away from the substrate. Thus, the conformational changes of Y204 in Xyl10E_A160E also altered the hydrophobic interactions between protein and ligand and consequently the substrate affinity and catalytic efficiency.

**Fig 4:**
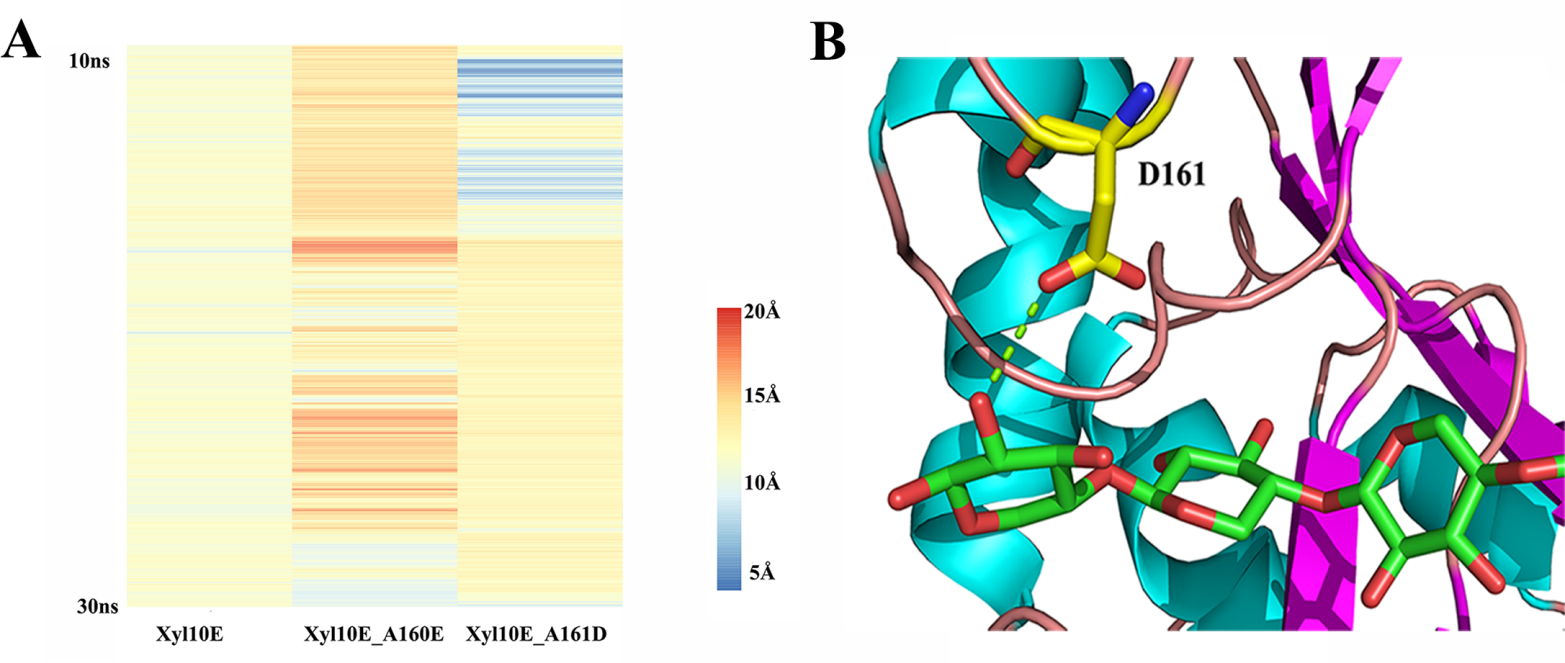
Analysis of the putative interactions of residue 161 and substrate. (A) The distance heatmap of Xyl10E and its mutants between the C_β_ of residue 161 and substrate. (B) The modelled hydrogen bond formed by D161 for binding substrate to the active site of Xyl10E_A161D.

The distances between the atom C_β_ of A161 or D161 and the substrate (abbreviated as 161_dist hereafter) were also calculated based on the MD results. According to the distance heatmap (Fig 4A), the 161_dist of Xyl10E was quite different from that of mutants Xyl10E_A160E and Xyl10E_A161D. This difference might be caused by the longer side chains of E160 and D161. Especially for A160E, the longer chain may affect the substrate binding (Fig 3) and result in the higher 161_dist values of Xyl10E_A160E. The 161_dist of Xyl10E_A161D changed a lot, revealing the dynamic relationship between D161 and substrate along with the swinging of loop 4 (low 161_dist value means substrate gets close to residue 161, and high value means substrate drifts away; Fig 4A). When loop 4 of the mutant Xyl10E_A161D got close to the substrate, a hydrogen bond formed between A161D and the substrate (Fig 4B), which was confirmed by the MD simulation results and reduced *K*_*m*_ value. As a result, the substrate affinity of Xyl10E_A161D was enhanced. When loop 4 swung upward and out of the catalytic pocket, D161 pulled the product away altogether. This behaviour promotes the release of the product and causes the *k*_*cat*_ value to increase.

## Discussion

A hyperthermophilic GH10 xylanase was identified from *Bispora* sp. MEY-1 with an impressive temperature optimum of 93–95°C although *Bispora* sp. MEY-1 has an optimal growth temperature of 28°C. This temperature optimum is significantly higher than most thermophilic xylanases of GH10 which have an optimal temperature of 60–70°C [13, 14]. To the best of our knowledge, only a few xylanases show hyperthermophilic characteristics with temperature optima at 80°C or higher [11, 20, 27]. For example, the XynB from *Pyrodictium abyssi* has the highest optimum temperature (up to 110°C) known so far [16], and the XynA from an environment sample demonstrates a temperature optimum of 100°C [28]. Compared to all known GH10 fungal xylanases that have a temperature optimum of 60 to 85°C [11–14, 20], Xyl10E may represent the most thermophilic xylanase from fungi, as Xyl10E has an optimum temperature at 93–95°C and maintains approximately 20% activity in boiling water.

Although Xyl10E showed the highest activity at pH 4.0, as many GH10 counterparts do [11, 12, 20], it showed high tolerance to a broader acidic pH range. When incubated at pH 1.0–7.0, 37°C for 1 h, it still retained more activity (Fig 1C) than *Gt*Xyn10 and *Tl*Xyn10A in acidic conditions [11, 12]. After treatment at pH 2.0–5.0, 70°C for 1 h, the enzyme was able to retain more than 90% activity. This excellent property makes Xyl10E suitable for biomass digestion in diluted acid and in high temperature conditions [29]. Moreover, the thermostability of Xyl10E was excellent compared to its counterparts, including *Mp*Xyn10A, which became inactive at 80°C for 1 h [27]; Xyl10g, which lost almost all activity at 70°C for 5 min [30]; and *Cb*Xyn10C, which had a temperature optimum at 85°C but lost activity at 90°C for 10 min [31]. Namely, Xyl10E showed high stability and activity at extremely high temperatures. Besides, Xyl10E displayed strong resistance to all tested metal ions and chemical reagents and may be suitable for biomass degradation in an ionic solution [32]. The sensitivity of Xyl10E to SDS is unrelated to thermostability [33], and its activity improvement by β-mercaptoethanol is similar to that of other GH10 xylanases [11, 12, 20].

Acidophilic and hyperthermophilic Xyl10E can be applied in several fields, but its catalytic efficiency (Table 2) was much lower than that of other GH10 xylanases, such as *Tl*Xyn10A (1,626 mL/s/mg) [11], Xyl10C (8,388 mL/s/mg) [20], *Au*Xyn10A (3,447 mL/s/mg) [34] and more. Therefore, the catalytic efficiency of Xyl10E must be improved for industrial application. It is generally considered that catalytic efficiency could be elevated by decreasing the *K*_*m*_ values or simultaneously reducing the *K*_*m*_ and increasing the *k*_*cat*_ values [19, 35–37]. In this study, mutagenesis of A160 and A161 to E or D on loop 4 improved the catalytic efficiency of Xyl10E through decreasing the *K*_*m*_ values or increasing the *k*_*cat*_ values, suggesting that mutagenesis at these two adjacent sites may employ different mechanisms to improve the catalytic efficiency. Our results also indicate that decreasing *K*_*m*_ through mutations may not always be a straightforward way to improve the catalytic efficiency.

In the present study, the MD simulation demonstrated that the distance between D161 and the substrate changed along with the swinging of loop 4. Possibly, when D161 got close to the product and formed a hydrogen bond, the loop swing helped to release the product, thus showing similar effects to that of the thumb loop of GH11 xylanases (which promotes product release with an upward motion) [38]. This conjecture has been supported by the increased *V*_*max*_ value. As A160 and A161 are located on loop 4 of Xyl10E near the +1 and +2 binding sites, and their mutagenesis causes enhanced substrate affinity or product release, it is obvious that loop 4 plays key roles in enzyme catalysis [39]. From the MD simulation of Xyl10E_A160E and Xyl10E_A161D, the residues A160E and A161D were presumed to affect the catalytic efficiency using a different mechanism. In other words, E160 changed the conformation of Y204 to increase the substrate affinity, while D161 bound the substrate with a hydrogen bond to promote the ligand capturing and product release as the thermal motion of loop 4. The amino acid frequency computed by the HotSpot Wizard indicated that the residue at position 160 should be Asn (68.5%), Glu (8%), Asp (7.5%), Ala (7.5%) and Ser (6.5%), and the residue at position 161 should be Asp (66.5%), Glu (23%), Ser (4%) and Gly (3%), which indicated that the counterpart of residue 161 in most of the GH10 xylanases could be involved in substrate binding or product release. As a thermostable xylanase with improved catalytic efficiency, Xyl10E demonstrates high potential for applications in bioenergy [7, 8], preparing animal feed [9], mashing [10] and other industries that use high-temperature biocatalysis.

## Conclusion

A hyperthermophilic xylanase of GH10 was identified in *Bispora* sp. MEY-1 and expressed in *P*. *pastoris* GS115. It demonstrated maximum activity at 93–95°C and pH 4.0. Residue replacement of A160 or A161 on loop 4 with E or D was capable of enhancing the catalytic efficiency up to 1.8-fold. The substitution A160E may change the conformation of the substrate binding site, Y204 (subsite +1), by forming an extra hydrogen bond and then increasing the substrate affinity, while the A161D is able to directly bind the substrate by forming a hydrogen bond, thus contributing to ligand trapping or accelerating the product release. This study not only provides a hyperthermophilic fungal xylanase and its mutants for research and industrial application but also sheds new insights on the roles of loop regions in enzyme catalysis.

## Supporting information

S1 Fig Chromatogram profile of the purification procedure of recombinant Xyl10E (HiTrap Q Sepharose). Blue line: UV280 absorbance; green line: the concentrate of elution buffer; brown line: electrical conductivity; and red arrow: the peak stands for Xyl10E.
(TIF)

S2 Fig SDS-PAGE analysis of Xyl10E and its mutants. Lanes: M, the molecular mass standards; 1, the crude enzymes; 2, the purified recombinant enzymes; 3, the deglycosylated enzymes with Endo H treatment.
(TIF)

S3 Fig Multiple sequence alignment of Xyl10E and other GH10 representatives. Residues A160 and A161 are indicated with red arrows.
(TIF)

S1 Table Primers used in this study. (DOC)

S2 Table Purification of the recombinant Xyl10E. (DOC)
