# Supplementary figures and images for: Improvement of the catalytic efficiency of a hyperthermophilic xylanase from Bispora sp. MEY-1
Source: PLoS One. 2017 Dec 18;12(12):e0189806. doi: 10.1371/journal.pone.0189806 (PMC5734778; doi:10.1371/journal.pone.0189806)

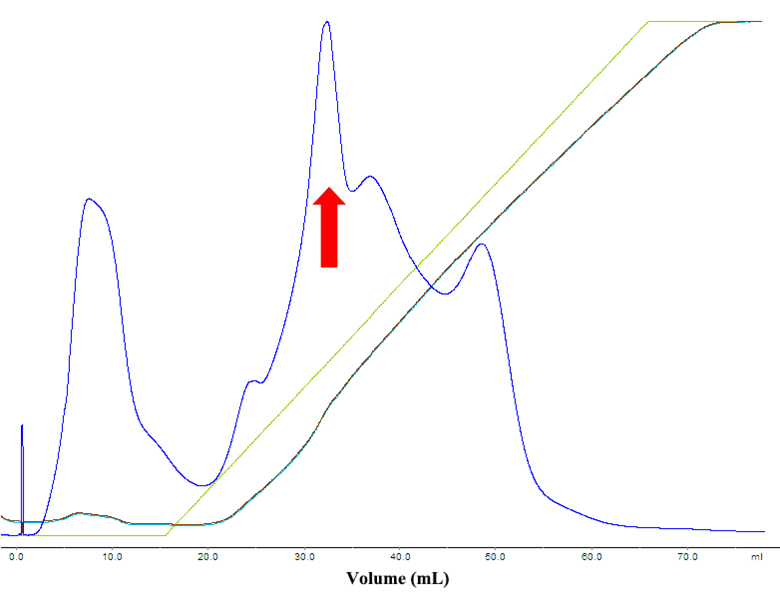

Supplement: S1 Fig — Blue line: UV280 absorbance; green line: the concentrate of elution buffer; brown line: electrical conductivity; and red arrow: the peak stands for Xyl10E. (TIF) [file pone.0189806.s001.tif]

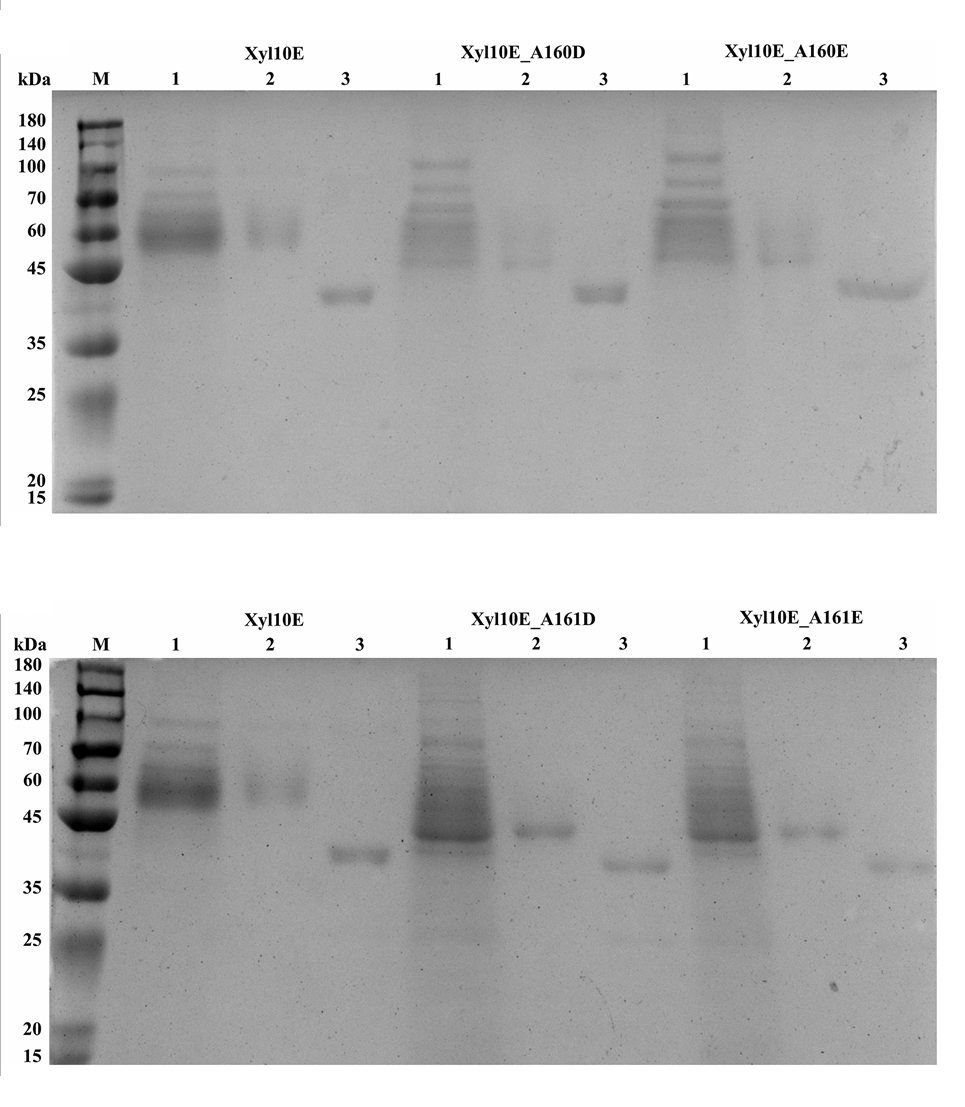

Supplement: S2 Fig — Lanes: M, the molecular mass standards; 1, the crude enzymes; 2, the purified recombinant enzymes; 3, the deglycosylated enzymes with Endo H treatment. (TIF) [file pone.0189806.s002.tif]

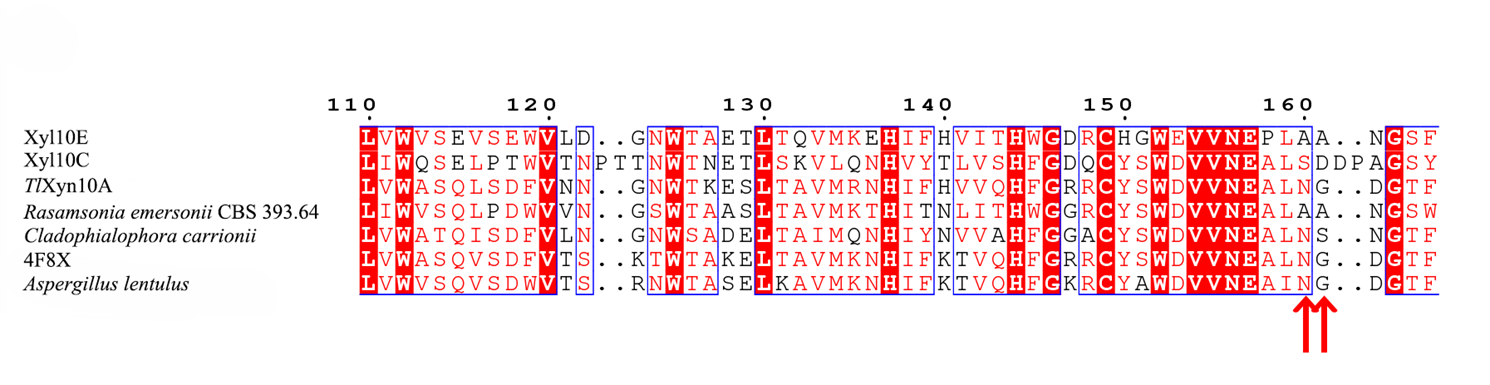

Supplement: S3 Fig — Residues A160 and A161 are indicated with red arrows. (TIF) [file pone.0189806.s003.tif]
