# Supplementary material for: Improvement of the catalytic efficiency of a hyperthermophilic xylanase from Bispora sp. MEY-1
Source: PLoS One. 2017 Dec 18;12(12):e0189806. doi: 10.1371/journal.pone.0189806 (PMC5734778; doi:10.1371/journal.pone.0189806)
Supplement: S1 Table — (DOC) [file pone.0189806.s004.doc]

**S1 Table.** Primers used in this study.

| Primers | Sequences (5′→3′)^a^ | |
| --- | --- | --- |
| Xyl10EF | | ATGCATCTGTCGTCGAGAATCGTG |
| Xyl10ER | | TCAAGTCGAGCAGACGGAACATG |
| Xyl10ESnabF | | ACTACGTAGGGCCGGTCTCGTCCGCTG |
| Xyl10ENotR | | TAAAGCGGCCGCTCAAGTCGAGCAGACGGAACATGGAATC |
| A160DF | | GAGCCGCTCGACGCGAACG |
| A160DR | | CGTTCGCGTCGAGCGGCTC |
| A160EF | | GAGCCGCTCGAGGCGAACG |
| A160ER | | CGTTCGCCTCGAGCGGCTC |
| A161DF | | CTCGCCGACAACGGATCGTTTG |
| A161DR | | CAAACGATCCGTTGTCGGCGAG |
| A161EF | | CTCGCCGAGAACGGATCGTTTG |
| A161ER | | CAAACGATCCGTTCTCGGCGAG |

^a^ The restriction sites are underlined
