# Supplementary material for: Improvement of the catalytic efficiency of a hyperthermophilic xylanase from Bispora sp. MEY-1
Source: PLoS One. 2017 Dec 18;12(12):e0189806. doi: 10.1371/journal.pone.0189806 (PMC5734778; doi:10.1371/journal.pone.0189806)
Supplement: S2 Table — (DOC) [file pone.0189806.s005.doc]

**S2 Table. Summary of the recombinant Xyl10E purification.**

| Purification step | Protein concentration (μg/mL) | | Total activity (U) | Specific activity (U/mg) | | Recovery rate (%) | |
| --- | --- | --- | --- | --- | --- | --- | --- |
| Crude | 95 | 11200 | | | 393 | | - |
| Vivaflow | 763 | 11600 | | | 453 | | 104 |
| Anion exchange | 178 | 4646 | | | 522 | | 41 |
